# Supplementary material for: Multimodal imaging and functional analysis of the chick NMDA retinal damage model
Source: PLoS One. 2021 Sep 7;16(9):e0257148. doi: 10.1371/journal.pone.0257148 (PMC8423281; doi:10.1371/journal.pone.0257148)
Supplement: S1 File — (ZIP) [file pone.0257148.s004.zip › SD-OCT/C200727/017/D15-PWk3/C200727--017_OS_R_14_0_REGAVG0000049/Manual_PreDefineThreeByFourGridRadialRetina_1.pdf]

Three By Four Grid Radial Retina Template Report

**Patient ID**	C200727	**Name**	See InVivoVue
**Exam Date**	See InVivoVue	**Exam Time**	See InVivoVue
**Eye**	OS	**Averaged**	N/A
**Depth (mm)**	1.65	**Depth Samples**	1024
**Pitch (um/pix)**	14.000 x 2333.333 x 1.608	**Refractive Index**	See InVivoVue
**Scan Type**	Radial 14.000mm x 14.000mm x 1.647mm , 1000 x 6 x 1024		

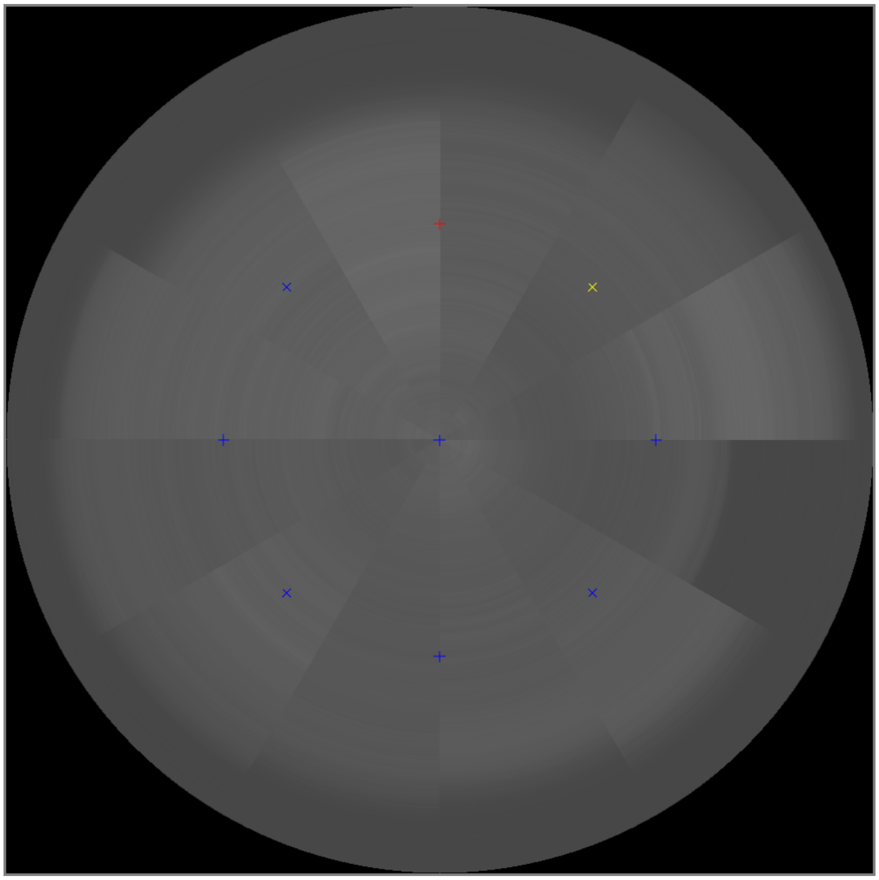

Thickness Metrics

Analysis	Average(mm)	Minimum(mm)	Maximum(mm)	STDEV(mm)
**IRNFL - RPE**	0.2734	0.2634	0.2964	0.0109
**IRNFL - ORNFL**	0.0381	0.0206	0.0755	0.0201
**ORNFL - OPL**	0.1555	0.1204	0.1966	0.0252
**OPL - ETPRS**	0.0620	0.0574	0.0643	0.0029
